# Supplementary material for: High-performance piezoelectric energy harvesting in amorphous perovskite thin films deposited directly on a plastic substrate
Source: Nat Commun. 2024 May 16;15:4129. doi: 10.1038/s41467-024-48551-3 (PMC11099020; doi:10.1038/s41467-024-48551-3)
Supplement: Supplementary file 1 — Supplementary Information [file 41467_2024_48551_MOESM1_ESM.pdf]

Supplementary Information

High-Performance Piezoelectric Energy Harvesting in Amorphous Perovskite Thin Films Deposited Directly on a Plastic Substrate

Ju Han, Sung Hyun Park, Ye Seul Jung, and Yong Soo Cho

Department of Materials Science and Engineering, Yonsei University, Seoul 03722, Korea

Correspondence should be addressed to Y.S.C. (email: ycho@yonsei.ac.kr)

Supplementary Table 1. Atomic percentage and relative ratios in amorphous CCTO thin films, which were estimated from the XPS spectra of Fig. 1c.

pO_2		Ca	Cu	Ti	O
1.8 mTorr	at.%	5.03	14.92	20.38	59.67
	Ratio	1.00	2.97	4.05	11.86
3.0 mTorr	at.%	5.02	14.88	20.18	59.92
	Ratio	1.00	2.98	4.04	11.98
4.0 mTorr	at.%	5.00	15.05	19.97	59.98
	Ratio	1.00	3.01	3.99	12.00

Supplementary Note 1. Procedure for the calculation of bending strain, s_b.

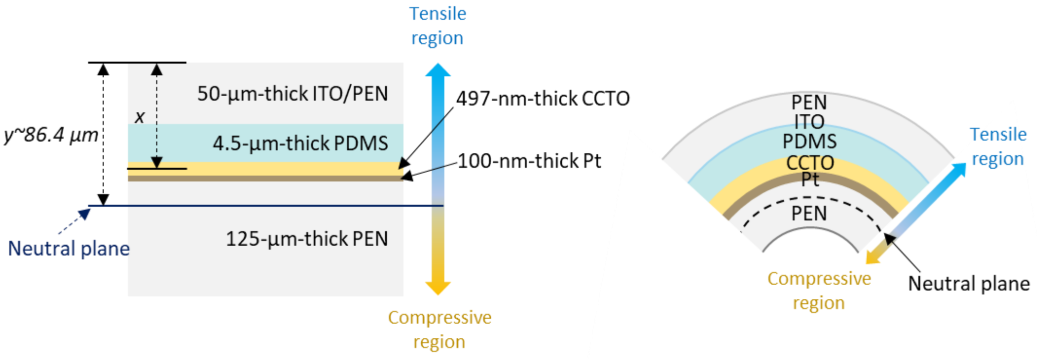

Supplementary Fig. 1. Estimation of the neutral plane in the harvester structure of PEN/ITO/PDMS/CCTO/Pt/PEN, considering their elastic moduli and thickness. Please refer to the following note for more details on the procedure for estimating the neutral plane and bending strain during bending operation.

After obtaining the position of the neutral plane in the six-layered harvester structure of PEN/ITO/PDMS/CCTO/Pt/PEN, we calculated the actual strain applied to the CCTO layer by considering the distance from the top of the harvester to the CCTO layer, as described below.

1) Estimation of the neutral plane

The location of the neutral plane, y, relative to the top surface was calculated by considering the contribution of each layer using the following equation reported for a multi-layered composite structure (*Science* **325**, 977-981 (2009)):

$$y = \frac{\sum_{i=1}^{6} E_i^* t_i \left(\sum_{j=1}^{i} t_j - \frac{t_i}{2}\right)}{\sum_{i=1}^{6} E_i^* t_i} \quad (1)$$

where $E_i^* = E_i/(1-\nu_i)$ (here, E_i and ν_i are Young's modulus and Poisson's ratio of the *ith* layer, respectively), and t_i and t_j are the thicknesses of the *ith* and *jth* layers, respectively.

The following data were used for the calculation:

$E_1 = E_6$ = 6.1 GPa, $\nu_1 = \nu_7$ = 0.33 for the 50- and 125-μm-thick PEN substrates (*J. Soc. Inf. Disp.* **15**, 1075-1083 (2007))
E_2 = 100 GPa, ν_2 = 0.2 for the 300-nm-thick ITO electrode (*Thin Solid Films* **460**, 156-166 (2004))
E_3 = 1.1 MPa, ν_3 = 0.45 for the 4.5-μm-thick PDMS layer (*J. Micromech. Microeng.* **17**, 623 (2007))
E_4 = 256 GPa, ν_4 = 0.25 for the 497-nm-thick CCTO thin film (*Mater. Lett.* **64**, 1226-1228 (2010))
E_5 = 141 GPa and ν_5 = 0.38 for the 100-nm-thick Pt layer (*Acta Mater.* **66**, 370-377 (2014))

The neutral plane *y* was found to be 86.4 μm below the top surface for the 497-nm-thick CCTO layer.

2) Calculation of the bending strain in the harvester

The bending strain s_b applied in the CCTO layer was calculated using the relation $s_b = (y-x)/r$, where *x* is the distance from the top of the harvester to the middle of the CCTO layer, and *r* is the radius of the bending curvature. The subsequent bending strain values were finally attained with respect to the magnitude of the bending curvature.

Supplementary Table 2. Calculated values of the radius of curvature and bending strain.

Radius of curvature (mm)	Bending strain (%)
7.03	0.45
5.74	0.55
4.97	0.63
4.44	0.71
4.06	0.77

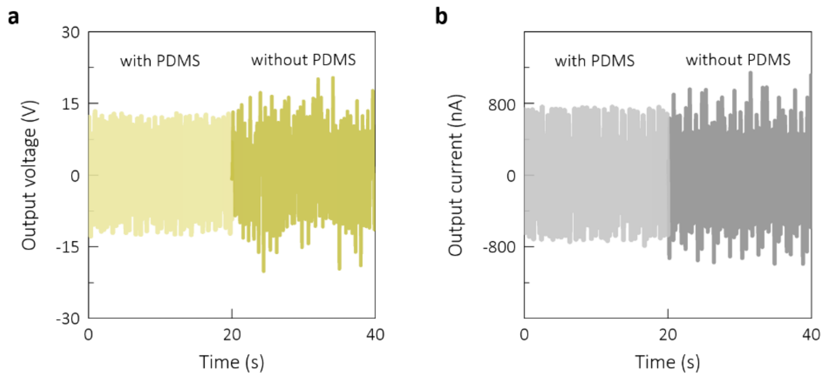

Supplementary Fig. 2. Comparison of output performance, **a** voltage and **b** current, of the harvesters with and without the intermediate PDMS layer. The CCTO layer was deposited at 3.0 mTorr.

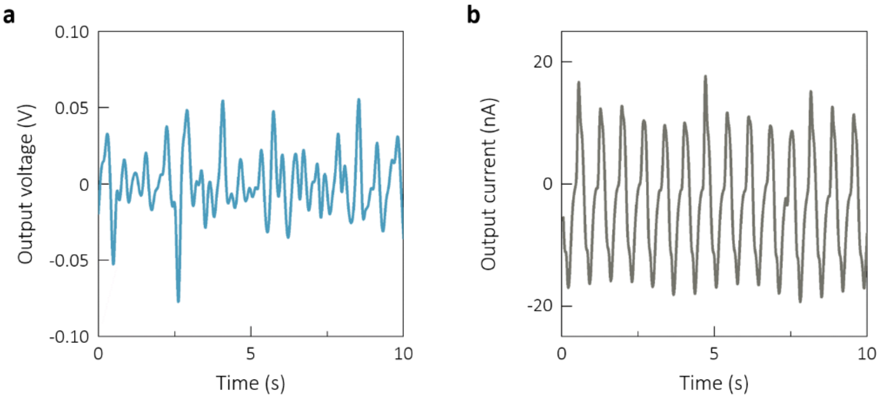

Supplementary Fig. 3. a Output voltage and **b** output current measured with only the PDMS layer (i.e., without the CCTO film), indicating the trivial contributions by the PDMS layer.

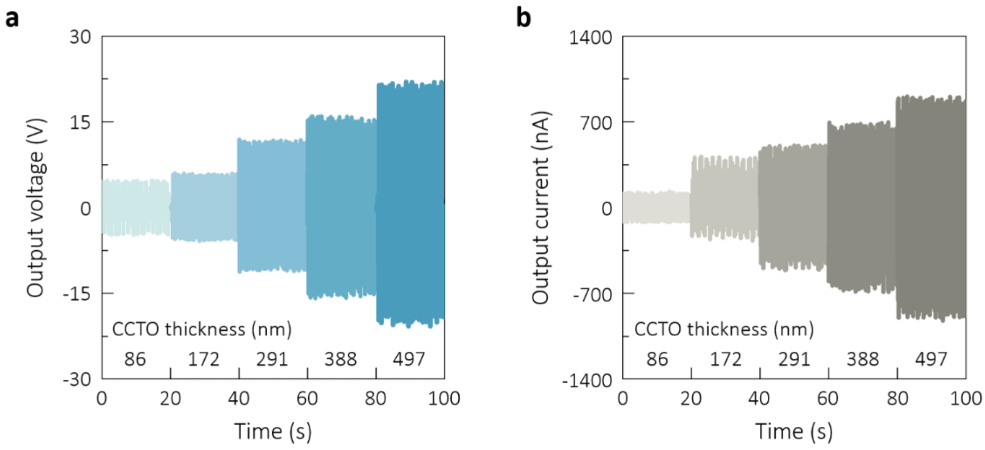

Supplementary Fig. 4. **a** Output voltage and **b** output current for the CCTO thin films having different film thickness, which were measured under the optimal bending strain of 0.77% and bending frequency of 3.10 Hz.

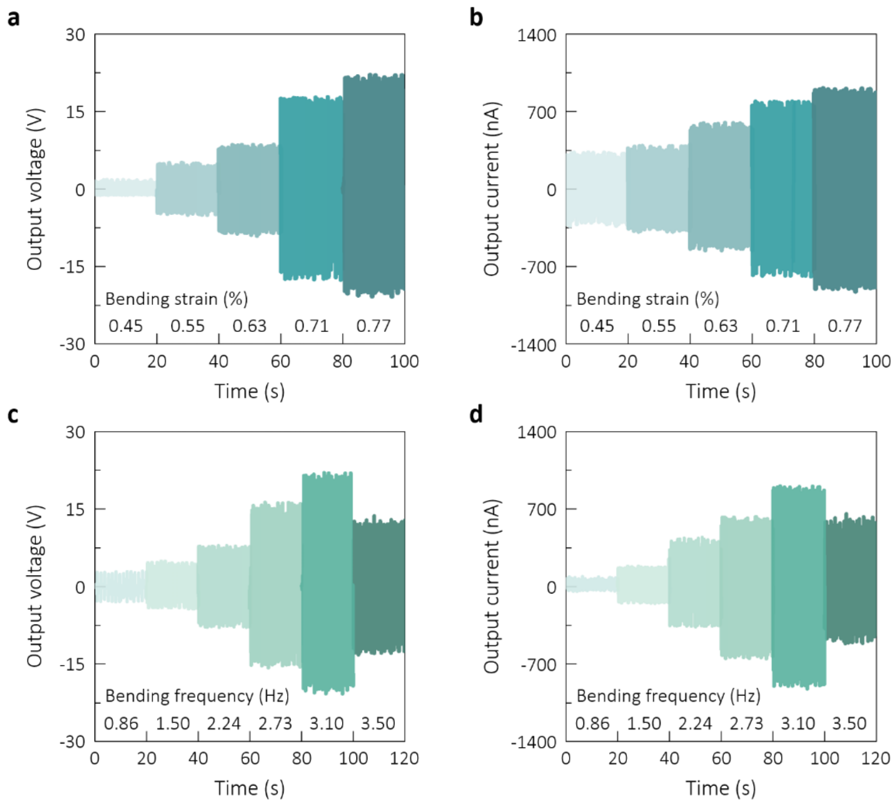

Supplementary Fig. 5. Piezoelectric energy-harvesting performance for the ~497-nm-thick CCTO thin films deposited at 4.0 mTorr: **a** Output voltage and **b** output current measured at a fixed frequency of 3.10 Hz with the bending strain increasing from 0.45% to 0.77%. **c** Output voltage and **d** output current measured at a bending strain of 0.77% with the bending frequency increasing from 0.86 to 3.50 Hz.

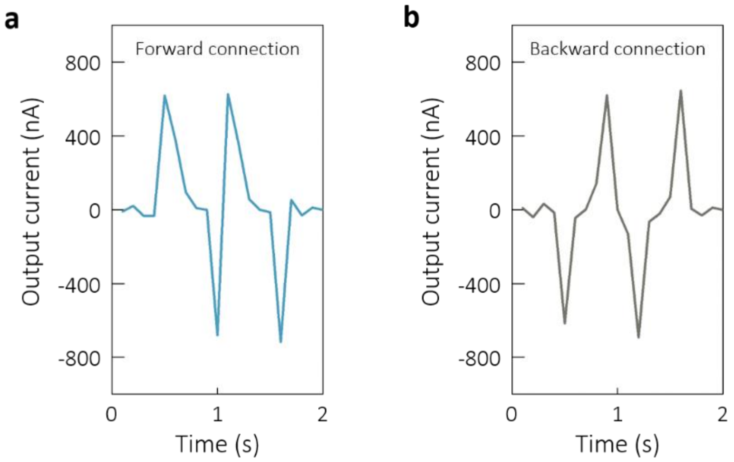

Supplementary Fig. 6. Polarity-switching behavior with **a** forward and **b** backward connections, suggesting that the output voltages were generated by the piezoelectric CCTO layer.

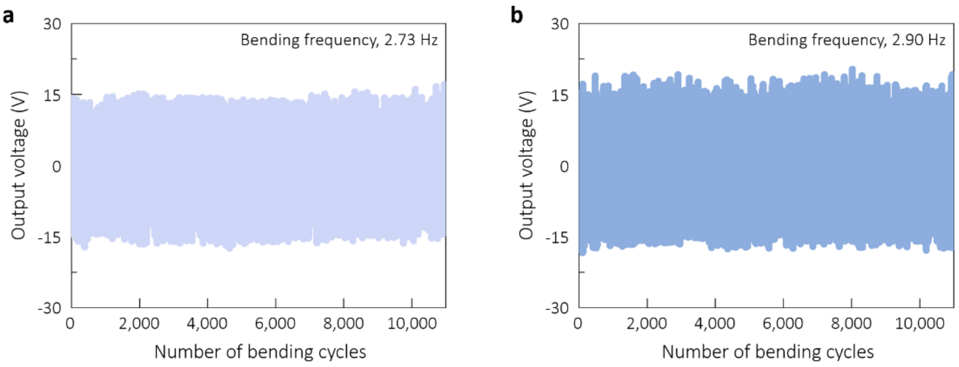

Supplementary Fig. 7. Stability evaluation of the output voltage over 11,000 cycles for the CCTO thin film deposited at 4.0 mTorr, measured at the bending strain of 0.77 % for two bending frequencies, **a** 2.73 and **b** 2.90 Hz.

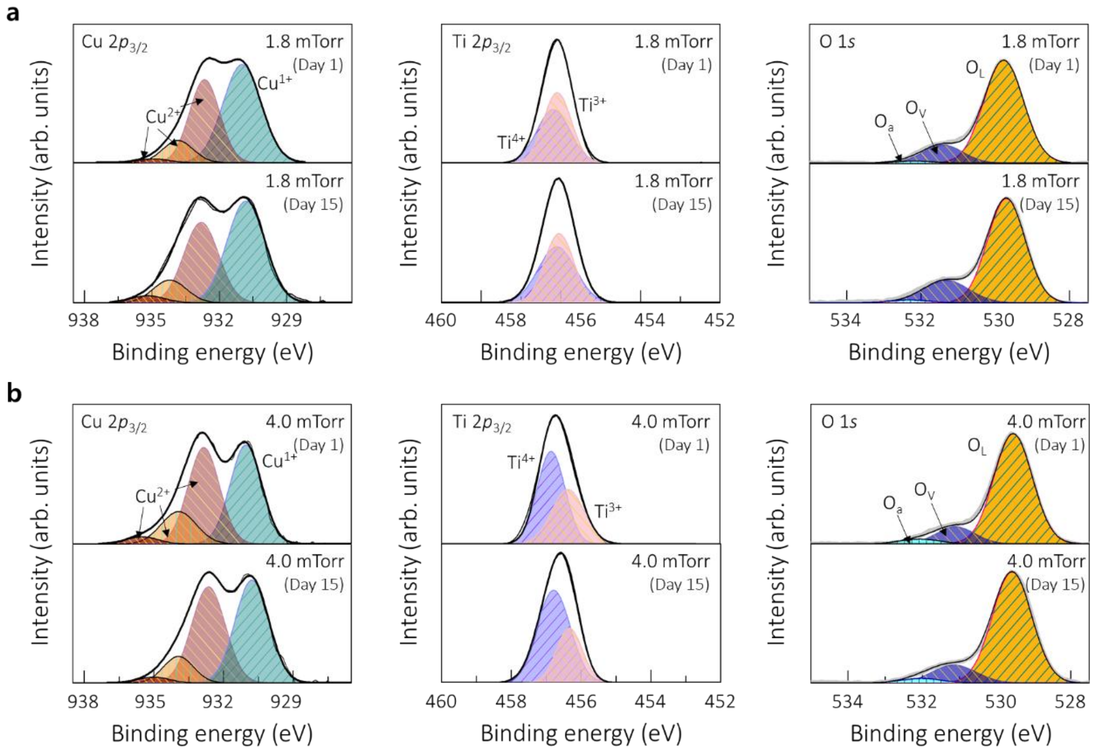

Supplementary Fig. 8. XPS spectra of the CCTO thin films deposited at **a** 1.8 and **b** 4.0 mTorr after 15 days' exposure in ambient atmosphere (relative to the case of the first day) for the chemical states of Cu 2$p_{3/2}$, Ti 2$p_{3/2}$, and O 1s.

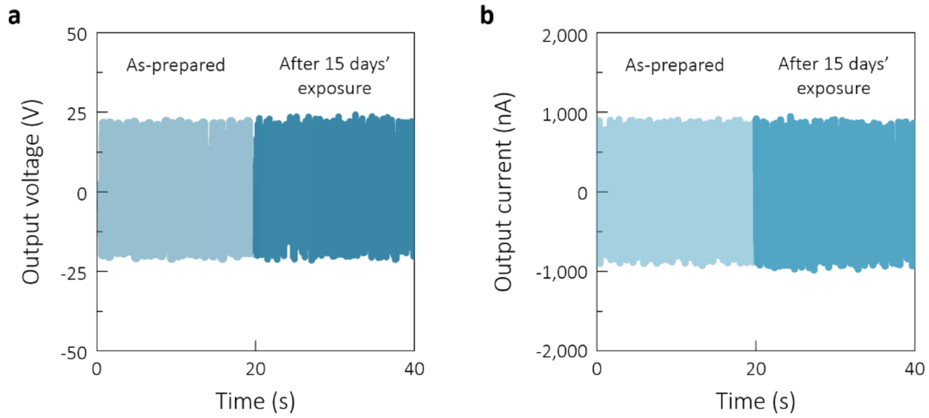

Supplementary Fig. 9. Comparison of output performance, **a** voltage and **b** current, of the 4.0 mTorr harvesters after 15 days' exposure in ambient atmosphere.

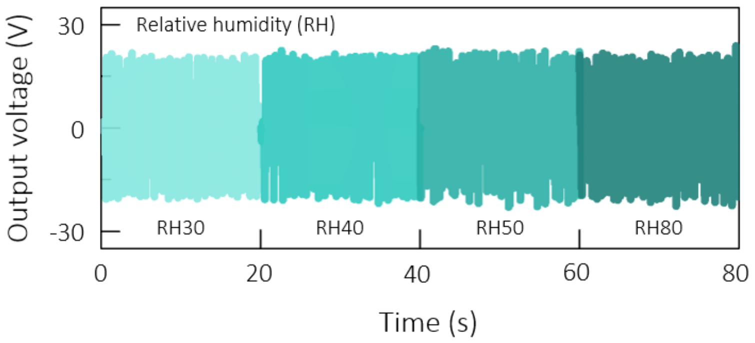

Supplementary Fig. 10. Output voltage generated by the optimal CCTO thin-film harvester with the change in humidity levels, which were measured at the bending strain of 0.77 % and bending frequency of 2.90 Hz.

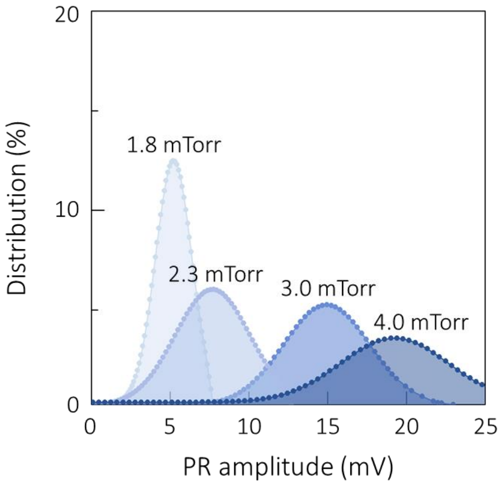

Supplementary Fig. 11. Distribution of PR amplitudes for the CCTO films deposited at different oxygen partial pressures, which were measured by PFM.

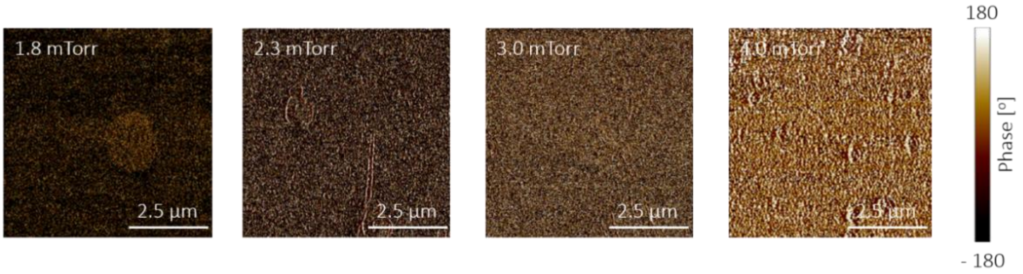

Supplementary Fig. 12. PR phase images of the ~497-nm-thick CCTO thin films deposited at different oxygen partial pressures.

Supplementary Table 3. Estimation of the piezoelectric voltage coefficient g_{33} and the figure of merit (FOM) for the harvesters based on amorphous CCTO thin films processed at different oxygen partial pressure. Note that we used the dielectric constant values measured at 40 kHz to match with the PFM frequency.

Oxygen partial pressure	d_{33} (pm V^{-1})	ε_r ($\varepsilon_{33}/\varepsilon_o$)	g_{33} (m V N^{-1})	FOM (10^{-12} m^2 N^{-1})
1.8 mTorr	1.73	5	0.039	0.06
2.3 mTorr	5.16	12	0.049	0.25
3.0 mTorr	17.8	38	0.052	0.94
4.0 mTorr	27.7	71	0.044	1.22

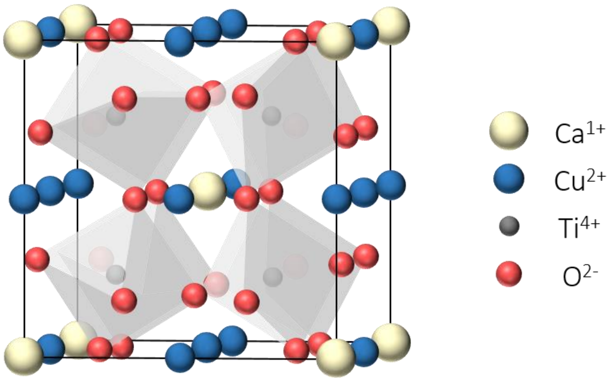

Supplementary Fig. 13. Crystal structure of stoichiometry CCTO unit cell.

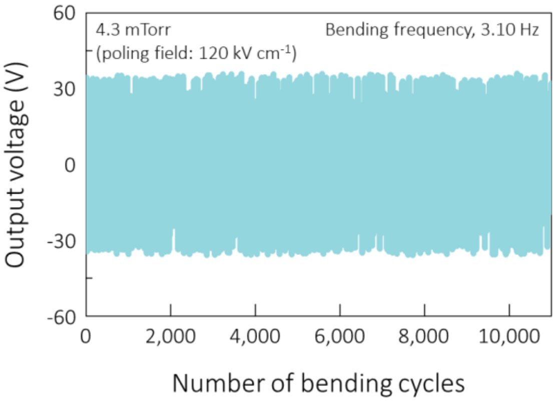

Supplementary Fig. 14 Stability evaluation of the output voltage over 11,000 cycles for the 4.3 mTorr sample poled at 120 kV cm^{-1}, measured at the bending strain of 0.77% and the bending frequency of 3.10 Hz.

Supplementary Table 4. Comparison of our best harvesting performance with representative results reported for piezoelectric thin-film harvesters based on various piezoelectric materials, such as perovskite oxides, ZnO, AlN, and perovskite halides.

Material (Substrate)	Deposition method	Film thickness (nm)	Poling field (kV cm^{-1})	Output voltage (V)	Output current (μA)	Power (μW)	Power density (μW cm^{-3})	Mechanical input source (condition)	Ref. (Ref. in main text)
$Pb(Zr_{0.53}Ti_{0.47})O_3$ (Stainless steel)	Sputtering	2,800	Poled	2.6	N/A	244	1.1×10^6	Vibration (50 Hz)	1 ([3])
$Pb(Zr,Ti)O_3$ (Si)	Spin-coating	1,000	Poled	0.16	N/A	2.15	3.2×10^3	Vibration (462 Hz)	2 ([10])
Mn-$Pb(Zr_{0.52}Ti_{0.48})O_3$ (Ni foil)	Sputtering	3,000	Poled	2.44	N/A	60	1.6×10^6	Vibration (72 Hz)	3 ([11])
Mn-$(K_{0.5}Na_{0.5})NbO_3$ (Si)	Spin-coating	1,000	120	0.52	N/A	3.6	1.8×10^3	Vibration (132 Hz)	4 ([63])
$(K,Na)NbO_3$	Sputtering	2,200	Poled	0.42	N/A	1.6	4.1×10^2	Vibration (393 Hz)	5 ([64])
AlN (Si)	Sputtering	500	No poling	2.48	N/A	20.5	4.1×10^3	Vibration (210 Hz)	6 ([66])
$(Bi_{0.5}Na_{0.5})TiO_3$ -$BaTiO_3$ (Si)	Spin-coating	2,000	600	0.75	N/A	2.22	4.6×10^4	Vibration (42 Hz)	7 ([8])
PZT (Stainless steel)	Sputtering	3,000	Poled	9.4	N/A	13.4	5.3×10^4	Pressing (finger)	8 ([62])
ZnO (PET)	Sputtering	2,000	No poling	2.25	N/A	0.28	1.4×10^3	Vibration (370 Hz)	9 ([65])
AlN (PI)	Sputtering	900	No poling	0.7	N/A	1.4×10^{-3}	4.0×10^2	Bending (N/A)	10 ([67])
$CsPbBr_3$ (ITO-PEN)	Spin-coating	545	35	22.6	1.13	21.3	5.6×10^4	Bending (strain, 0.67%)	11 ([68])
$CsSnI_3$ (ITO-PEN)	Spin-coating	354	17.4	9.5	0.45	4.23	2.4×10^4	Bending (N/A)	12 ([69])
$MAPbI_3$ (ITO-PEN)	Spin-coating	486	11.3	23.1	1.70	182	2.7×10^5	Bending (strain, 0.47%)	13 ([70])
MA_2SnCl_6 (ITO-PET)	Spin-coating	300	50	12	1.20	7.33	2.4×10^5	Pressing (0.5 MPa)	14 ([71])
Amorphous $CaCu_3Ti_4O_{12}$ (PEN)	Sputtering	497	120	38.7	1.24	413	2.8×10^6	Bending (strain, 0.77%)	This work

(PZT: $Pb(Zr,Ti)O_3$, MA: methylammonium, PI: polyimide, PET: polyethylene terephthalate, PEN: polyethylene naphthalate)

Supplementary Table 5. Comparison of our best harvesting performance with representative results reported for piezoelectric filler-polymer matrix composite harvesters.

Filler (content)	Polymer (substrate)	Thickness (μm)	Poling field (kV cm^{-1})	Output voltage (V)	Output current (μA)	Power (μW)	Power density (μW cm^{-2})	Power density (μW cm^{-3})	Mechanical Input sources (condition)	Ref. (Ref. in main text)
PZN-PZT (30 vol.%)	UV polymer (ITO-PET)	15	75	2.96	0.36	0.81	0.17	1.1 x 10^2	Bending (strain, 0.85%)	15 ([72])
$BaTiO_3$ (10 wt.%)	PVDF (ITO-PET)	40	500	102* (peak to peak)	10* (peak to peak)	280	70	1.8 x 10^4	Pressing (500 kPa)	16 ([73])
$BaTiO_3$ (30 wt.%)	PDMS (Cu foil)	180	300	3.05	2.5	1	0.25	1.7 x 10^2	Pressing (-)	17 ([74])
$NaNbO_3$ (1 vol.%)	PDMS (PS)	100	80	3.2	0.07	27	6	6.0 x 10^2	Bending (strain, 0.23%)	18 ([75])
$BiFeO_3$ (40 wt.%)	PDMS (PET)	100	200	3	0.3	0.25	0.12	1.2 x 10^1	Pressing (finger)	19 ([76])
$CsPbBr_2I$ (10 wt.%)	PDMS (Polyester)	10	700	65	12	375	32.2	3.2 x 10^4	Pressing (1.3 N)	20 ([77])
$FAPbBr_3$ (35 wt.%)	PDMS (ITO-PET)	150	50	8.5	3.4	36	12	8.0 x 10^2	Pressing (0.5 MPa)	21 ([78])
$FAPbBr_3$ (12 wt.%)	PVDF (PET)	120	50	30	29.7	131	27.4	2.3 x 10^3	Pressing (0.5 MPa)	22 ([79])
$FASnBr_3$ (20 wt.%)	PDMS (ITO-PET)	80	750	94.6* (peak to peak)	19.1* (peak to peak)	118	18.9	2.4 x 10^3	Pressing (4.2 N)	23 ([80])
$MASnI_3$ (N/A)	PVDF (PI)	5	60	12	4	21.6	21.6	4.3 x 10^4	Pressing (0.5 MPa)	24 ([81])
$MASnBr_3$ (15 wt.%)	PDMS (PI)	172	55	18.8	13.76	74.5	74.5	4.3 x 10^3	Pressing (0.5 MPa)	25 ([82])
$(ATHP)_2PbBr_2Cl_2$ (30 wt.%)	PDMS (ITO-PET)	300	200	90* (peak to peak)	6.5* (peak to peak)	N/A	1.7	5.7 x 10^1	Pressing (4.2 N)	26 ([83])
$FAPbBr_2I$ (20 wt.%)	PVDF (Polyester)	30	40	85* (peak to peak)	30* (peak to peak)	144	10	3.3 x 10^3	Pressing (1.3 N)	27 ([84])
$TMCM_2SnCl_6$ (18 wt.%)	PDMS (Cu foil)	300	150	81	2	N/A	N/A	N/A	Pressing (4.9 N)	28 ([85])
$MAPbI_3$ (25 vol.%)	PVDF (ITO-PET)	98	80	45.6	4.7	N/A	N/A	N/A	Pressing (50 N)	29 ([86])
Amorphous $CaCu_3Ti_4O_{12}$	- (PEN)	(0.497)	120	38.7	1.24	413	138	2.8 x 10^6	Bending (strain, 0.77%)	This work

(PZN-PZT: $Pb(Zn_{1/3}Nb_{2/3})O_3$–$Pb(Zr_{0.5}Ti_{0.5})O_3$, UV: ultraviolet, FA: formamidinium, MA: methylammonium, ATHP: 4-aminotetrahydropyran, TMCM: trimethylchloromethylammonium, PS: polyester, PET: polyethylene terephthalate, PI: polyimide, PVDF: polyvinylidene fluoride, PDMS: polydimethylsiloxane)

*based on peak-to-peak values: half of each value needs to be considered for comparison with the other values in this Table.

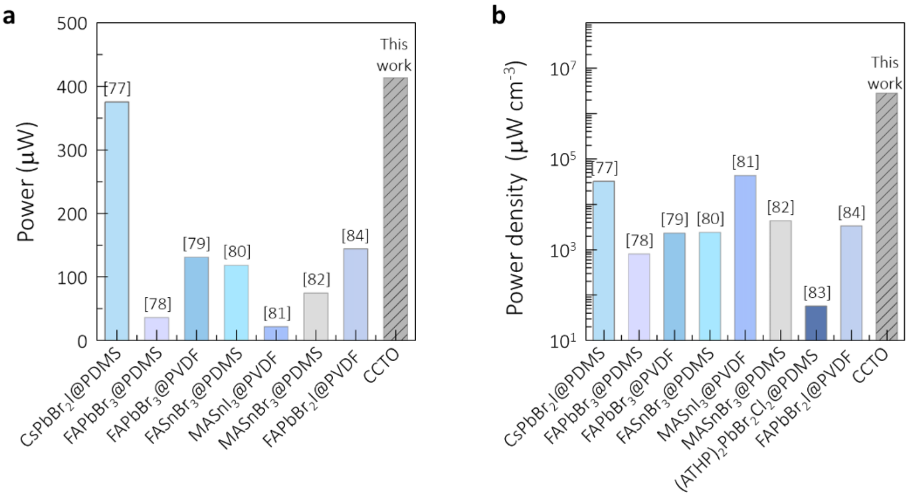

Supplementary Fig. 15. a Power and **b** power density values reported for halide-polymer composite harvesters, which are compared with our best outcomes.

Supplementary References

1. Morimoto, K., Kanno, I., Wasa, K. & Kotera, H. High-efficiency piezoelectric energy harvesters of c-axis-oriented epitaxial PZT films transferred onto stainless steel cantilevers. *Sens. Actuator A Phys.* **163**, 428–432 (2010).
2. Shen, D. et al. The design, fabrication and evaluation of a MEMS PZT cantilever with an integrated Si proof mass for vibration energy harvesting. *J. Micromech. Microeng.* **18**, 055017 (2008).
3. Yeo, H. G. & Trolier-McKinstry, S. Effect of piezoelectric layer thickness and poling conditions on the performance of cantilever piezoelectric energy harvesters on Ni foils. *Sens. Actuator A Phys.* **273**, 90–97 (2018).
4. Won, S. S. et al. Lead-free Mn-doped $(K_{0.5}Na_{0.5})NbO_3$ piezoelectric thin films for MEMS-based vibrational energy harvester applications. *Appl. Phys. Lett.* **108**, 232908 (2016).
5. Tsujiura, Y. et al. Lead-free piezoelectric MEMS energy harvesters of $(K,Na)NbO_3$ thin films on stainless steel cantilevers. *Jpn. J. Appl. Phys*. **52**, 09KD13 (2013).
6. Jia, Y. & Seshia, A. A. Power optimization by mass tuning for MEMS piezoelectric cantilever vibration energy harvesting. *J. Microelectromech. Syst.* **25**, 108–117 (2015).
7. Cho, A., Kim, D. B. & Cho, Y. S. Electric-field-dependent surface potentials and vibrational energy-harvesting characteristics of $Bi(Na_{0.5}Ti_{0.5})O_3$-based Pb-free piezoelectric thin films. *ACS Appl. Mater. Interfaces* **11**, 13244–13250 (2019).
8. Harada, R. Iwamoto, N., Kweon, S. H., Umegaki, T. & Kanno, I. Finger flexion power generators made of piezoelectric lead zirconate titanate thin films on stainless steel foils. *Sens. Actuator A Phys.* **322**, 112617 (2021).
9. Pan, C. T., Liu, Z. H., Chen, Y. C. & Liu, C.F. Design and fabrication of flexible piezo-microgenerator by depositing ZnO thin films on PET substrates. *Sens. Actuator A Phys.* **159**, 96–104 (2010).
10. Guido, F. et al. AlN-based flexible piezoelectric skin for energy harvesting from human motion. *Microelectron. Eng.* **159**, 174–178 (2016).
11. Kim, D. B., Jo, K. S., Park, S. J. & Cho, Y. S. Contribution of anisotropic lattice-strain to piezoelectricity and electromechanical power generation of flexible inorganic halide thin films. *Adv. Energy Mater*. **12**, 2103329 (2022).
12. Kim, D. B., Park, K. S., Park, S. J. & Cho, Y. S. Microampere-level piezoelectric energy generation in Pb-free inorganic halide thin-film multilayers with Cu interlayers. *Nano Energy* **92**, 106785 (2022).
13. Kim, D. B., Jo, K. S., Park, K. S. & Cho, Y. S. Anion-dependent polarization and piezoelectric power generation in hybrid halide $MAPbX_3$ (X = I, Br, and Cl) thin films with out-of-plane structural adjustments. *Adv. Sci.* **10**, 2204462 (2023).
14. Ippili, S. et al. Halide double perovskite-based efficient mechanical energy harvester and storage devices for self-charging power unit. *Nano Energy* **107**, 108148 (2023).
15. Kim, D. B., Kim, S. W., Kim, Y. E., Choi, H. J. & Cho, Y. S. Room-temperature processed $Ag/Pb(Zn_{1/3}Nb_{2/3})O_3$–$Pb(Zr_{0.5}Ti_{0.5})O_3$-based composites for printable piezoelectric energy harvesters. *Compos. Sci. Technol.* **218**, 109151 (2022).
16. Li, H., Lee, H. B., Kang, J. W. & Lim, S. Three-dimensional polymer-nanoparticle-liquid ternary composite for ultrahigh augmentation of piezoelectric nanogenerators. *Nano Energy* **113**, 108576 (2023).
17. Luo, C. et al. A flexible lead-free $BaTiO_3$/PDMS/C composite nanogenerator as a piezoelectric energy harvester. *Energy Technol*. **6**, 922–927 (2018).
18. Jung, J. H. et al. Lead-free $NaNbO_3$ nanowires for a high output piezoelectric nanogenerator. *ACS Nano* **5**, 10041–10046 (2011).
19. Ren, X., Fan, H., Zhao, Y. & Liu, Z. Flexible lead-free $BiFeO_3$/PDMS-based nanogenerator as piezoelectric energy harvester. *ACS Appl. Mater. Interfaces* **8**, 26190–26197 (2016).
20. Khan, A. A. et al. Control of halogen atom in inorganic metal-halide perovskites enables large piezoelectricity for electromechanical energy generation. *Small* **19**, 2303366 (2023).
21. Ding, R. et al. Flexible piezoelectric nanocomposite generators based on formamidinium lead halide perovskite nanoparticles. *Adv. Funct. Mater*. **26**, 7708–7716 (2016).
22. Ding, R. et al. High-performance piezoelectric nanogenerators composed of formamidinium lead halide perovskite nanoparticles and poly(vinylidene fluoride). *Nano Energy* **37**, 126–135 (2017).
23. Rana, M. M. et al. Enhanced piezoelectricity in lead-free halide perovskite nanocomposite for self-powered wireless electronics. *Nano Energy* **101**, 107631 (2022).
24. Ippili, S. et al. An eco-friendly flexible piezoelectric energy harvester that delivers high output performance is based on lead-free $MASnI_3$ films and $MASnI_3$-PVDF composite films. *Nano Energy* **57**, 911–923 (2019).
25. Ippili, S., Jella, V., Kim, J., Hong, S. & Yoon, S. G. Unveiling predominant air-stable organotin bromide perovskite toward mechanical energy harvesting. *ACS Appl. Mater. Interfaces* **12**, 16469–16480 (2020).
26. Khan, A. A. et al. Superior transverse piezoelectricity in organic-inorganic hybrid perovskite nanorods for mechanical energy harvesting. *Nano Energy* **86**, 106039 (2021).
27. Khan, A. A. et al. Maximizing piezoelectricity by self-assembled highly porous perovskite–polymer composite films to enable the internet of things. *J. Mater. Chem. A* **8**, 13619–13629 (2020).
28. Huang, G. et al. Achieving ultrahigh piezoelectricity in organic–inorganic vacancy-ordered halide double perovskites for mechanical energy harvesting. *ACS Energy Lett*. **6**, 16–23 (2021).
29. Jella, V., Ippili, S., Eom, J. H., Choi, J. & Yoon, S. G. Enhanced output performance of a flexible piezoelectric energy harvester based on stable $MAPbI_3$-PVDF composite films. *Nano Energy* **53**, 46–56 (2018).
